# Vaccines and Senior Travellers

**DOI:** 10.3389/fragi.2021.677907

**Published:** 2021-07-09

**Authors:** Fiona Ecarnot, Stefania Maggi, Jean-Pierre Michel, Nicola Veronese, Andrea Rossanese

**Affiliations:** ^1^ University Hospital Besancon and University of Franche-Comté, Besancon, France; ^2^ CNR, Institute of Neuroscience – Aging Branch, Padua, Italy; ^3^ Department of Rehabilitation and Geriatrics, University of Geneva, Geneva, Switzerland; ^4^ Geriatrics Section, Department of Medicine, University of Palermo, Palermo, Italy; ^5^ Department of Infectious-Tropical Diseases and Microbiology, IRCCS “Sacro Cuore-Don Calabria,” Verona, Italy

**Keywords:** older adults, vaccines, travel, immunization, seniors 65 and over

## Abstract

**Background:** International tourist travel has been increasingly steadily in recent years, and looks set to reach unprecedented levels in the coming decades. Among these travellers, an increasing proportion is aged over 60 years, and is healthy and wealthy enough to be able to travel. However, senior travellers have specific risks linked to their age, health and travel patterns, as compared to their younger counterparts.

**Methods:** We review here the risk of major vaccine-preventable travel-associated infectious diseases, and forms and efficacy of vaccination for these diseases.

**Results:** Routine vaccinations are recommended for older persons, regardless of whether they travel or not (e.g., influenza, pneumococcal vaccines). Older individuals should be advised about the vaccines that are recommended for their age group in the framework of the national vaccination schedule. Travel-specific vaccines must be discussed in detail on a case-by-case basis, and the risk associated with the vaccine should be carefully weighed against the risk of contracting the disease during travel. Travel-specific vaccines reviewed here include yellow fever, hepatitis, meningococcal meningitis, typhoid fever, cholera, poliomyelitis, rabies, Japanese encephalitis, tick-borne encephalitis and dengue.

**Conclusion:** The number of older people who have the good health and financial resources to travel is rising dramatically. Older travellers should be advised appropriately about routine and travel-specific vaccines, taking into account the destination, duration and purpose of the trip, the activities planned, the type of accommodation, as well as patient-specific characteristics, such as health status and current medications.

## Introduction

The number of older travellers is on the rise. The World Tourism Organization estimates that tourist arrivals increased by 4% in 2019, to reach a record 1.5 billion, continuing a trend that has been evident over the last decade (United Nations World Tour, 2021). Many of these travellers are older individuals, and although age in itself is not a contra-indication to travel, there are specificities in older persons that merit consideration in the context of travel. The natural, age-related decline in physiological function known as immunosenescence means that the body is more susceptible to external stressors, and less well able to mount an adequate immune response in case of infection. This phenomenon affects both innate (natural) and adaptive (acquired) immunity and is characterized by two main features, namely an impaired ability to respond to new antigens, and an impaired ability to sustain memory response after infection (Goronzy and Weyand, 2013). Consequently, older people may be at higher risk of infection, and more likely to experience a severe and/or complicated course of disease. Vaccine responses in older individuals often fail to generate sufficient protection and exhibit reduced efficacy (Allen et al., 2020). For some vaccines, it has been shown that immune responses develop more slowly in older adults [e.g., in hepatitis A (Van Der Meeren et al., 2015) and hepatitis B (Weinberger et al., 2018), among others (Weinberger et al., 2010)]. Therefore, the time between administration of the vaccine and the start of travel is of particular importance for older adults. In parallel, a faster decline in antibody titers is observed with increasing age (Weinberger et al., 2008). It also makes a difference whether primary (first) or booster vaccinations are applied. Kaml et al. demonstrated that booster vaccinations against tetanus, pertussis and diphtheria resulted in a less robust response in subjects over 65 years of age compared to younger individuals, whereas the immune response to all three components of the vaccine was better in those older patients who had been previously vaccinated against Tdap (Kaml et al., 2006). This reinforces the concept of regular booster vaccinations throughout life (Weinberger et al., 2008). There may also be a background of frailty or chronic disease, which raise their own challenges in the context of travel, particularly if traveling to areas where the healthcare system is of a lower standard.

Several diseases that pose a risk to travellers may be effectively prevented by vaccination. However, changing immune response and the presence of comorbid conditions may change the risk-benefit ratio of certain vaccines. We review the risk of major vaccine-preventable travel-associated diseases, and the forms and efficacy of vaccination for these diseases.

## Routine Vaccination and Travel

Irrespective of travel plans, the vaccine status of older individuals should regularly be monitored and updated, during routine contact with healthcare professionals. Elderly individuals should be advised about the vaccines that are recommended, available and (in many countries) reimbursed for their age group in the framework of the national vaccination schedule. The person’s status with regard to diphtheria–tetanus–pertussis (DTP), measles–mumps–rubella (MMR) and herpes zoster vaccination should be investigated, and where possible, recorded in a personal health record, and the patient advised to receive initial vaccination (if not previously immunized) or booster doses, as appropriate. Tetanus in particular is of special relevance for persons planning to travel. Indeed, in a study performed among 331 adults aged 18–60 years attending travel vaccine clinics in France and who had all been vaccinated according to national recommendations, Launay et al. reported that the percentage of subjects with antibodies to tetanus and diphtheria decreased with age (Launay et al., 2009). Similarly, they also reported that the mean time since the last booster dose increased with age, to reach over 9 years (9.4 years, 95% CI, 6.8–12.1) in those aged 50 to 60. In addition, they found that the total number of diphtheria vaccine (primary vaccination and boosters) doses received was significantly lower in older individuals (*p* <0.001), and only 62.7% (95% CI 50.8–74.6) of those aged 50–60 years had anti-diphtheria antibodies at a level considered to be seroprotective (i.e., ≥0.1 IU/ml). Weinberger et al. also reported that recall response to diphtheria and tetanus vaccination was insufficient in older individuals in their study of 252 persons aged above 60 years who received a booster vaccination against tetanus and diphtheria, and a subgroup (*n* = 87) that received a second booster 5 years later (Weinberger et al., 2013). Although almost all participants had protective antibody concentrations at 4 weeks after the first vaccination, antibodies fell below levels considered to be seroprotective in 10% (for tetanus) and 45% (for diphtheria) after 5 years. Protection was restored in almost all vaccinees after the second vaccination (Weinberger et al., 2013). These findings underline the need for regular and well-documented booster shots of recommended vaccines in adults throughout the life course, independently of travel plans. This is of particular importance in view of the latest epidemiological data regarding diphtheria, showing an upward trend in the number of cases worldwide 2017, after a steady decline from 2000 to 2016, suggesting that progress in the fight against diphtheria worldwide has stalled (Clarke et al., 2019). The WHO South-East Asia region has reported the majority of global diphtheria incidence each year since 2000, and within the WHO South-East Asia region, India, Nepal, and Indonesia together accounted for 96–99% of cases (Clarke et al., 2019). Population migration or political instability in certain regions likely contributed to creating conditions favorable to outbreaks, with the largest most recent outbreaks reported in the Rohingya refugee population in Bangladesh, and in Yemen and Venezuela (Clarke et al., 2019).

### Influenza Vaccination

Routine influenza vaccination should be advised and administered annually. In the European Union, all countries recommend the influenza vaccine for individuals aged over 65 years, independently of travel. According to the most recent log scale diagram by Steffen (2018), influenza is the most incident vaccine-preventable disease in travellers. Due to physiological immune-senescence, vaccine efficacy among older adults is significantly lower (37% in those aged 65 years and older vs. 51% in those aged 18–64 years) (Belongia et al., 2016). These numbers have also been confirmed by the Canadian Serious Outcome Surveillance (SOS) Network, although the authors also demonstrated that, despite this rather low efficacy, the number of deaths prevented by the influenza vaccine is around 75% for those aged 65 years and older (Nichols et al., 2018). There are different vaccines available in different parts of the world. For example, there are separate vaccines for the Northern and Southern hemispheres, although their strains are often similar (Lambach et al., 2015). There is a live-attenuated intranasal influenza vaccine, not available in Europe, which is not recommended for people aged 65 or older (Grohskopf et al., 2018). There are several licensed vaccines for seniors, i.e., a trivalent inactivated high-dose vaccine, a quadrivalent inactivated adjuvanted vaccine and a quadrivalent recombinant vaccine, particularly suitable for those with an egg allergy (Grohskopf et al., 2018). A high-dose quadrivalent inactivated influenza vaccine (HD-IIV4; Fluzone High-Dose Quadrivalent, Sanofi Pasteur) was licensed by the Food and Drug Administration in 2019 for the prevention of influenza in persons aged 65 years and older (Chahine, 2021). In a phase III trial in the United States, it was shown to be well tolerated and to induce non-inferior immune responses compared to those induced by the trivalent high-dose vaccine for the shared strains (Chang et al., 2019). The preferred vaccine is the adjuvanted formula, because the presence of MF59 enhances the response by the senescing immune system through the recruitment and activation of immune cells at the injection site. In addition, MF59-adjuvanted vaccine was shown to induce a stronger booster response than the non-adjuvanted vaccine, and also provided broader serological protection against drifted strains that circulated in the 2 years after vaccination (Ansaldi et al., 2008). MF59 has also been shown to induce local stimulation and recruitment of dendritic cells (DCs), and subsequent increased uptake of antigen by DCs (Coffman et al., 2010). Through this increased recruitment of immune cells, facilitating migration of DCs into tissue-draining lymph nodes, the MF59 adjuvant helps to prime adaptive immune responses (Seubert et al., 2008). Finally, in a head-to-head comparison of standard-dose and enhanced vaccines among 1861 adults aged 65–82 years, Cowling et al. reported that recipients of enhanced vaccines showed improved humoral and cell-mediated immune responses compared to those receiving standard-dose vaccines (Cowling et al., 2020).

The major issue when deciding whether or not to give an influenza shot to a senior traveler is that the vaccine is usually not available in summer. Hence, two practical possibilities arise for travellers leaving at the end of winter for the opposite hemisphere: 1) vaccinate (or re-vaccinate) with the vaccine of the home hemisphere relying on cross-reactivity, and 2) obtain the vaccine of the destination hemisphere soon after arrival (Vaccines for travelers. M, 2018). There is no formal preference expressed by the Advisory Committee for Immunisation Practices (ACIP) and no delay is allowed, should a specific product be unavailable (Grohskopf et al., 2018).

### Pneumococcal Vaccine

Among the routine vaccinations that providers should be recommending and administering to older individuals, perhaps the most important with regard to travel is the pneumococcal vaccine. *Streptococcus pneumoniae* is a ubiquitous pathogen that causes a large spectrum of invasive and non-invasive disease in both children and adults. Pneumococcal pneumonia remains the most common form of invasive pneumococcal disease (IPD), and it disproportionately affects older individuals and those with weakened immune systems, such as patients with human immunodeficiency virus or chronic disease (Berical et al., 2016; Rivero-Calle et al., 2019). Other severe types of disease, such as meningitis or septicaemia may also occur, while less severe presentations such as acute otitis media or sinusitis are common in children. *Streptococcus pneumoniae* is reportedly responsible for 30–38% of community-acquired pneumonia (Johansson et al., 2010; Holter et al., 2015). Hospital-acquired pneumonia is among the most frequent nosocomial infections, and is associated with prolonged hospital stay and high costs (Guidelines for the manage, 2005), and the case fatality rate increases in line with increasing age (Dirmesropian et al., 2019). *Streptococcus pneumoniae* is spread by coughing, sneezing and respiratory secretions, and up to 50% of children may be asymptomatic carriers (Hussain et al., 2005), although carriage rates decrease to around 10% in adulthood (Goldblatt et al., 2005), and even lower in elderly individuals (Ridda et al., 2010). The incidence of IPD in 2013/2014 was reported to be 6.85/100,000 for all adult age groups and 20.58/100,000 among individuals aged >65 years in the United Kingdom (Thomas, 2021), while there were an estimated 192,281 hospital admissions for pneumonia and 6,000 cases of IPD in 2013/14 (Chalmers et al., 2016). Data are sparse regarding the incidence of IPD specifically among travellers, but available data from studies in Hajj pilgrims have reported that *Streptococcus pneumoniae* accounted for six out of nine cases of meningitis from among 808 patients hospitalized in 2 hospitals in 2003 (Madani et al., 2006), 19% (12/64) of isolates in ICU patients with sepsis in two major hospitals in Makkah during the 2004 season (Baharoon et al., 2009), while in 2005, 2.7% (44/1,626) of isolates from patients with sepsis in four Mecca hospitals were due to *S. pneumoniae* (Asghar, 2006).

An increasing body of evidence in favor of drug resistance to a growing list of antibiotics, including penicillin, cephalosporins, macrolides and fluoroquinolones, underline the need for vaccines to be used widely to prevent pneumococcal disease. In elderly individuals who are traveling, often in enclosed spaces such as airplanes, ships or buses with high potential for transmission, vaccination against pneumococcal disease is therefore of paramount importance.

There are two pneumococcal vaccines currently available, namely the 23-valent pneumococcal polysaccharide vaccine (PPSV23), and the 13-valent pneumococcal conjugate vaccine (PCV13). PPSV23 is recommended for all adults aged 65 years and older, while PCV13 is widely recommended in infant vaccination schedules worldwide, and for adults aged 65 years and over. Both pneumococcal vaccines are recommended for adults aged less than 65 in the presence of immune-compromising conditions. The antibody response stimulated by PPSV23 is T-cell independent, leading to waning protection over time (Musher et al., 2010; Aliberti et al., 2014) and there is no induction of immunological memory (Rijkers et al., 2018). Therefore, booster shots should be recommended in line with recommended intervals.

There can be no doubt as to the efficacy of vaccination in preventing invasive and non-invasive pneumococcal disease. A systematic review and meta-analysis of the efficacy of PPSV23 in older adults reported pooled efficacy in 73% against invasive pneumococcal disease caused by any serotype (Falkenhorst et al., 2017). Similarly, in the CAPAMIS study, Ochoa-Gondar et al. reported that PPSV23 was effective in reducing the risk of all-cause and pneumococcal pneumonia (Ochoa-Gondar et al., 2014). For conjugate vaccines, the randomized, double-blind CAPITA trial including over 84,000 adults reported vaccine efficacy of 75% (95% CI 41.4–90.8%) for the prevention of a first episode of vaccine-type invasive pneumococcal disease with PCV13 (Bonten et al., 2015). A recent review of the literature found no evidence of hyporesponsiveness after repeat vaccination with PPSV23 at an interval of 5 or more years after the previous dose, or after sequential administration after PCV (Cripps et al., 2021).

Consequently, pneumococcal vaccination should be routinely recommended to older individuals, particularly when they plan to travel, and irrespective of whether the travel involves planned participation in mass gatherings. While there is no specific evidence to indicate that pneumococcal vaccination is especially necessary in healthy individuals planning to travel, travel consultations are a useful opportunity to recommend this vaccine, which should be a part of routine healthcare in all individuals over 65 years of age. The CDC recommendations for pneumococcal vaccination are summarized in Table 1.

**TABLE 1:** Centre for disease control recommendations for pneumococcal vaccination.

• For immunocompetent adults aged 65 years and older, vaccination with PCV13 is no longer routinely recommended, but may be used after shared clinical decision-making in those not previously vaccinated with PCV13
• For travellers in this age group, healthcare providers should discuss with patients the fact that PCV13 vaccine serotypes may still be circulating in some destination countries with lower vaccination rates
• All immunocompetent adults aged ≥65 years should receive 1 dose of PPSV23 ≥5 years after any previous PPSV23 dose, regardless of previous pneumococcal vaccine history
• No additional doses of PPSV23 should be given following the dose administered at age ≥65 years
• For immunocompetent adults aged 65 years and older, if a decision to give PCV13 has been made, 1 dose of PCV13 should be administered first, followed by 1 dose of PPSV23 ≥12 months after PCV13
• Immunocompetent adults aged 19 through 64 years with chronic medical conditions (e.g., chronic heart disease excluding hypertension; chronic lung or liver disease; diabetes, alcoholism, cigarette smoking) should receive a single dose of PPSV23
• For immunocompetent adults aged ≥65 years with chronic medical conditions (e.g., chronic heart disease excluding hypertension; chronic lung or liver disease; diabetes, alcoholism, cigarette smoking), vaccination with PCV13 is no longer routinely recommended, but may be used after shared clinical decision-making
• Immunocompetent adults aged ≥65 years with chronic medical conditions (e.g., chronic heart disease excluding hypertension; chronic lung or liver disease; diabetes, alcoholism, cigarette smoking) should receive 1 dose of PPSV23. If PCV13 has been given, then give PPSV23 ≥1 year after PCV13 and ≥5 years after any PPSV23 at age <65 years
• PCV13 and PPSV23 should NOT be administered during the same visit
• Pneumococcal vaccine can be administered concomitantly with seasonal influenza vaccine
• Contraindications to pneumococcal vaccine include notably previous hypersensitivity reaction to the vaccine or any component thereof

## Travel-Specific Vaccines

Travel-specific vaccines must be discussed in detail on a case-by-case basis, and the risk associated with the vaccine should be carefully weighed against the risk of contracting the disease during travel. Some destination countries may require vaccination against certain diseases before allowing entry, and this must be taken into consideration. Alternative itineraries could be envisaged if the vaccine requirements of the destination are not compatible with the traveler’s state of health.

### Yellow Fever

Yellow fever (YF) is a vector borne disease that is transmitted by the *Aedes* species of mosquitoes, and mainly a zoonotic disease in monkeys and apes, occasionally infecting humans. The majority of infections are asymptomatic, with a ratio of symptomatic to asymptomatic infection ranging from 1:7 to 1:12 (Vasconcelos and Monath, 2016), or may lead only to mild disease. However, a small proportion of patients will progress to the second phase of disease, characterised, as the name suggests, by haemorrhagic febrile jaundice. The virus is endemic in tropical areas of Africa and Central and South America. The overall estimated risk of acquiring YF for an unvaccinated traveler for a 2 weeks stay to a high-risk area in Africa is 50 cases per 100,000 people, and five cases per 100,000 people in South America (Reno et al., 2020). Between 2016 and 2018, there were several outbreaks of YF in various parts of the world (notably Brazil), resulting in increased numbers YF in travellers from affected areas (Reno et al., 2020).

YF vaccine is a live attenuated 17D virus, in use since the 1950s and grown on fertilized eggs. There is only one vaccine available, and it is known to be highly effective–a single dose of YF vaccine confers sustained immunity within 10 days for over 80% of those vaccinated, and within a month for almost all those vaccinated. It provides life-long protection against the disease. The question of whether or not to give YF vaccine is a dilemma, not only in older adults, but in all travellers. The World Health Organization (WHO) policy on YF vaccine and risk states that vaccination is recommended for all travellers aged 9 months or older traveling to areas where there is evidence of persistent or periodic YF virus transmission. Vaccination is generally not recommended for travellers going to areas where the risk of exposure is low and any risk should be weighed against individual risk factors (e.g., age, immune-status) for vaccine-associated adverse events, but a number of countries require incoming visitors to be vaccinated against YF (World Health Organization, 2019a).

The actual population risk of YF is hard to estimate. A recent study taking account of vaccination coverage estimated the YF burden in Africa for the year 2013 as 130,000 (95% CI 51,000–380,000) cases with fever and jaundice or haemorrhage, including 78,000 (95% CI 19,000–180,000) deaths (Garske et al., 2014). An outbreak of YF occurred in Luanda (Angola) in 2016 that rapidly spread to the Democratic Republic of Congo, and from there, was carried onwards by travellers to Mauritania, Kenya, and even China, the first time in history that confirmed YF cases have occurred in Asia. The tardy response and shortfall of YF vaccines led some experts to qualify YF as a threat to the entire world (Woodall and Yuill, 2016). These outbreaks, along with outbreaks in Brazil and Nigeria in 2017 prompted the WHO to introduce the Eliminate Yellow Fever (EYE) strategy, to respond to the increased threat of urban YF outbreaks with international spread. In absolute terms, with a reported 677 deaths from the 2 outbreaks in Brazil from 2016 (World Health Organization, 2018a), and 140 deaths for all of Africa in 2016 (Yellow fever in Africa an, 2017), one might consider the absolute risk for a traveler staying only a few days to weeks to be relatively low. The CDC estimates that for a 2-week stay, the estimated risk of infection and death from YF for an unvaccinated traveler visiting an endemic area in West Africa is 50 per 100,000 and 10 per 100,000, respectively; and 5 per 100,000 and 1 per 100,000, respectively in South America (Gershman and Staples, 2018). In practice, the actual risk is highly variable across regions, and even within countries. Guidance from vaccine recommendation maps is helpful, but may be based on historical data that no longer accurately reflect the real-time risk (Fletcher et al., 2020).

It has been shown that the neutralizing antibody response to YF vaccination is not diminished in healthy older adults, with equivalent categorical and quantitative responses across younger and older subjects in clinical trials involving a total of 4,532 subjects (Monath et al., 2005). Roukens et al. found that older subjects (aged 60–81 years) had a delayed antibody response and higher viraemia levels after yellow fever primovaccination compared to their younger counterparts (18–28 years) but there were only 28 subjects in the older group and 30 in the younger group in this study (Roukens et al., 2011).

YF vaccine was long considered to be one of the safest vaccines available, but in recent years, this has been called into question by the occurrence of two major types of adverse events following YF vaccination, namely YF vaccine-associated neurologic disease (YEL-AND) and YF vaccine-associated viscerotropic disease (YEL-AVD). YEL-AND may occur within 2–56 days post-vaccination (Fletcher et al., 2020) and may take the form of various distinct clinical syndromes, such as meningoencephalitis, Guillain-Barre syndrome (GBS), or acute disseminated encephalomyelitis (ADEM) (Lindsey et al., 2016). It is usually self-limiting, neurological sequelae are unusual and deaths are rare (1–2%) (Monath, 2012). Certain acquired risk factors are known to increase susceptibility to YEL-AND and are of relevance to older individuals considering YF vaccination, namely immune suppression and age >60 years. Indeed, immune suppression promotes prolonged viremia, and incurs a higher risk of neuroinvasion. Similarly, the age-related decline in immunity or the frequent presence of comorbidities (e.g., hypertension, diabetes) put older individuals at higher risk (Monath, 2012).

YEL-AVD is a rare, but serious, multisystemic disease with multiorgan failure, that mimics wild-type YF infection and is frequently fatal. It may occur up to 18 days after a first YF vaccination (Fletcher et al., 2020). The frequency of YEL-AVD is estimated at between 0 and 12 cases per 100,000 vaccinees (Seligman, 2014), but rates vary widely between regions, with a 33-fold higher rate in Peru and a 19-fold lower rate in Brazil as compared to estimates from the US Vaccine Adverse Event Reporting System (Seligman, 2014). The reporting rate of YEL-AVD increases with age (Lawrence et al., 2004; Lindsey et al., 2016). In a review of adverse events occurring after YF vaccination between 2007 and 2013, Lindsey et al. reported 6 cases of YEL-AVD after an estimated 2,224,790 doses of YF vaccine, corresponding to a reporting rate of 0.3; of note, 5 of these cases occurred in persons aged over 70 years (reporting rate = 4.0), and all were males (Lindsey et al., 2016). Using a list of cases from the US Centre for Disease Control (CDC), incremented by literature data, Seligman identified potential risk groups for YEL-AVD as older (>56 years) males, women between the ages of 19 and 34, people with various autoimmune diseases, individuals who have been thymectomized because of thymoma, and children under the age of 11 years (Seligman, 2014).

Despite intense research into the mechanisms that underpin these complications, our understanding of host genetics and the host immune response after vaccination remains incomplete. Pulendran et al. described the interaction of YF-17D with the innate immune system and the consequences of these interactions on the stimulation of the adaptive immune response (Pulendran, 2009). The application of systems biology approaches to studying immune responses shows that such systems approaches could be used to identify early signatures of vaccination that predict the immunogenicity and efficacy of vaccines. More recently, Azamor et al. conducted a follow-up study in volunteers immunized with YF 17D, investigating the humoral response, cellular phenotypes, gene expression, and selected single nucleotide polymorphisms, to clarify the role and interaction of these factors in early response after vaccination, and concluded that the early events elicited after YF 17D vaccination are, at least partly, associated with and controlled by human genetic background (Azamor et al., 2021).

Considering the overall absolute number of people at risk, and the low rate of serious vaccine-related adverse events and infection, the risk-benefit ratio of YF vaccination must be weighed carefully in travellers, especially those who are older in age. As a general rule, if the risk of getting YF is greater than the risk of serious adverse events, then vaccination should be mandatory, bearing in mind that YF vaccination is obligatory for access to certain countries. Personal factors, such as overall health status, genetic disposition, age or immune status, also play a role and should be taken into consideration in deciding on YF vaccination. Finally, travel-related factors, such as the destination, and the type of activity the person plans to undertake there, will also be important. Points to consider when deciding on the need for YF vaccination for travel are summarized in Table 2. Although it may be challenging to cover so many points in a short travel consultation, the best strategy is to present all the data to the patient and allow them to make their own decision, unless YF vaccine is obligatory for the country they plan to travel to. For persons who cannot get YF vaccine for medical reasons and planning to travel to a country with a YF vaccination entry requirement, the physician may provide a medical waiver letter and complete the medical contraindication section of the International Certificate of Vaccination or Prophylaxis.

**TABLE 2:** Points to consider when deciding on the need for yellow fever vaccination for travellers.

• Is YF fever mandatory for entry into the destination country or other countries on the itinerary?
• Has the traveler been previously immunised with YF?
• Current age
• Immunocompromised
• Presence of autoimmune disease
• Patient thymectomized
• Itinerary and activities on site
• Destination YF activity and transmission
• WHO assessment
• Travellers opinion, thoughts and consent on vaccination, based on the above

### Hepatitis

Hepatitis A is a very common travel-related infectious disease and one of the most common causes of viral hepatitis worldwide. Evaluation of the risk of a senior of contracting hepatitis A during travel must consider their destination, since the majority of touristic destinations in the world are places where hepatitis A is endemic, and there is likely to be a risk of travellers of any age encountering the virus. In a population-based study of the incidence rate of travel-related hepatitis A from 1997 to 2005, Askling et al. reported that the incidence rate in unprotected travellers to East Asia, North Africa, and the Middle East was 2, 12, and 18 cases/100,000 person-months, respectively, although children aged 0–14 years accounted for 43% of all travel-related cases (Askling et al., 2009).

Seroprevalence of anti-HAV immunoglobulin (Ig) G, a marker of prior infection with HAV, increases with age, and data from the United States collected before widespread implementation of HAV vaccination, indicate that 75% of individuals aged over 70 years had serological evidence of prior HAV infection (Bell et al., 2005), such that pre-vaccination screening for evidence of prior infection may be warranted. Development of anti-HAV IgG antibodies confers lifelong immunity against re-infection (Marcus and Tur-Kaspa, 1997). Older individuals are at greater risk of more severe disease. Indeed, in the elderly, the disease course tends to be prolonged, with more frequent jaundice, coagulopathy, cholestasis, pancreatitis and ascites (Marcus and Tur-Kaspa, 1997; Carrion and Martin, 2012). As a result, hospitalization, morbidity, and mortality rates increase with age (Marcus and Tur-Kaspa, 1997; Carrion and Martin, 2012; Nagaratnam et al., 2017). As for YF, immune status, travel destination, and activities on site should be kept in mind when considering the risk of hepatitis A in older people planning to travel. Indeed, older people have a lower risk of infection, given their higher likelihood of previous exposure, but they are at risk of greater clinical severity if infected compared with their younger counterparts. A review of studies investigating response to hepatitis A vaccination according to age reported lower seroconversion rates, and also that titers of antibody to hepatitis A virus achieved after vaccination were inversely proportional to age (Leder et al., 2001). In an open-label study, D’Acremont et al. compared the immune response of subjects aged 18–45 years old to a cohort over 50 years old and showed that the seroprotection rates were respectively 100 and 65% after the first vaccination, and 100 and 97% after the booster dose (D'Acremont et al., 2006). These findings show that hepatitis A is a well-tolerated and immunogenic vaccine in the older population.

Hepatitis B also has low endemicity in Europe, but as with hepatitis A, there can be some risk of exposure during travel, particularly in relation to sexual behaviors or medical/surgical procedures, notably due to accidents occurring during travel (Zuckerman and Steffen, 2000), and medical emergencies related to underlying chronic health conditions in the traveler. Medical tourism is also associated with an increased risk of infection. A meta-analysis by Anker et al. reported that patients traveling to undergo kidney transplant were more likely to develop hepatitis B virus infection compared to domestic kidney transplant recipients (Anker and Feeley, 2012). The hepatitis B virus (HBV) is highly prevalent, and despite widespread vaccination, a large proportion of the world population remains unprotected (Miglietta et al., 2018a). Universal vaccination programmes have been successful in reducing HBV infections in many countries (Zanetti et al., 2008). This success has been linked to four main criteria, namely initiation of universal vaccination before 1995, implementation of catch-up vaccination, achievement of high coverage rates, and endemicity, whereby countries starting from high endemicity saw a greater impact of vaccination (Miglietta et al., 2018a). A report of the trends in HBV infection over 5 decades in Italy found that sexual transmission and cosmetic treatment with percutaneous exposure such as piercing and tattooing, are the major modes of transmission nowadays (Sagnelli et al., 2014). The WHO Global Hepatitis Report 2017 indicates that the use of childhood HBV vaccination has greatly reduced the incidence of new chronic HBV infections, and most of those now living with such infections are persons who were born before the availability and implementation of childhood vaccination programmes (World Health Organization, 2017a). Steffen reported higher risk for persons working in endemic countries for prolonged periods, as compared to short-term vacationers (Steffen, 1990). In a European study of 9,008 individuals, 6.6–11.2% of travellers were classified as being at high risk of hepatitis B infection, depending on the destination country (Zuckerman and Steffen, 2000). In a pooled analysis of data from healthy adults aged ≥20 years who had been vaccinated with 20 μg HBV vaccine (Engerix™ B, GSK Vaccines, Belgium) in a 0, 1, 6 months schedule in 11 studies performed since 1996, Van Der Meeren et al. found a protection rate of 98.6% in the 20–24 years age group, which decreased to 64.8% in those aged ≥65 years (Van Der Meeren et al., 2015). In a study of 52 healthy, community dwelling elderly adults (65–82 years), seronegative for HBV, enrolled in the SENIEUR protocol, Edelman et al. reported that lower seroprotection rates were achieved in the healthy seniors overall compared to a junior cohort (aged 21–34 years), with delayed response to vaccination (achieving seroprotection only after the third dose), and lower peak titers (Edelman et al., 2020).

Regarding the recommendations for vaccination, a combined vaccine should be proposed if the traveler is at risk or likely to become at risk of both hepatitis A and hepatitis B; otherwise, a single vaccine can be proposed depending on the endemicity of the virus in the destination country. The eligibility of people with chronic health conditions for vaccination is often questioned, but it should be noted that it is highly recommended for persons with chronic comorbidities to receive hepatitis vaccination.

### Meningococcal Meningitis

Invasive meningococcal disease (IMD) is caused by the bacterium *Neisseria meningitidis* and predominantly affects infants and young children (Strifler et al., 2016; Marten et al., 2019). Although present worldwide, incidence of meningococcal disease is highest in the so-called “meningitis belt” in Sub-Saharan Africa, stretching from Senegal and Guinea across to Ethiopia (Pizza et al., 2020). The risk for travellers is highest among people visiting meningitis belt countries who have prolonged contact with local populations during an epidemic (Centers for Disease Contr, 2020a), and cases of meningococcal diseases in Hajj pilgrims to Saudi Arabia have also been reported (Pizza et al., 2020). Data regarding the true risk to travellers are rare, but the risk is generally acknowledged to be low and cases are rare (Memish, 2002), although the severity of the disease means that the danger should not be underestimated (Wilder-Smith, 2007).

The risk of contracting meningococcal meningitis (MM) during an individual travel is low, and it is just as relevant when considering vaccination needs in seniors to consider the person’s risk of getting the disease while at home. Over the period 2004–2014, serogroup B caused 74% of all notified cases of IMD in the EU/EEA, and the majority of cases in all age groups, whereas there were decreasing trends in serogroups B and C and an upward trend in serogroup Y (Whittaker et al., 2017). IMD remains rare at less than 1 case per 100,000 population in the EU/EEA (Whittaker et al., 2017), but in the event of an outbreak, disease risk can increase 1000-fold, and outbreaks can last up to 10 years or more (Oviedo-Orta et al., 2015). In Europe, the highest incidence was observed in the Republic of Ireland and the United Kingdom, while incidence has reached historically low levels in the United States in recent years (Parikh et al., 2020). In South America, Argentina, Chile and Brazil have the highest burden of IMD, although this is likely due to improved surveillance and laboratory testing available in these countries (Sáfadi et al., 2015; Parikh et al., 2020). Risk factors for IMD include viral infections, smoking, alcohol, crowded spaces, and health conditions such as immunodeficiency (e.g., HIV), hemoglobinopathy, asplenia or other chronic conditions such as diabetes and renal failure. Personal behaviors were found to be related to risk in a recent study of disease clusters occurring in Italy (Miglietta et al., 2018b).

A number of European countries recommend meningococcal vaccination for newborns and/or young children. Both vaccines should also be offered to high risk individuals, those with chronic conditions, or those engaging in high-risk personal behaviors. Available vaccines include a quadrivalent conjugate vaccine covering serogroups A, C, W, and Y, and a serogroup B meningococcal vaccine. It should be noted that there is waning immunity with meningococcal vaccines (Centers for Disease Contr, 2011). In a phase IIIb, open-label, randomized, controlled study, Dbaibo et al. evaluated the immunogenicity and safety of a meningococcal conjugate vaccine (MenACWY-TT) and a quadrivalent polysaccharide vaccine in adults aged over 55 years (range 56–103 years). They reported that one month after a single dose of vaccine, at least 97.4 and 95.5% of subjects in the conjugate and polysaccharide groups respectively had serum bactericidal antibodies above the cutoff indicative of seroprotection (Dbaibo et al., 2013). Vaccine response rates tended to be lower in subjects who were aged >65 years at the time of vaccination, compared to their younger counterparts (Dbaibo et al., 2013). In a study from Latin America, Stamboulian et al. reported that the quadrivalent meningococcal conjugate vaccine (MenACWY-CRM) was well tolerated and elicited higher immune responses compared to an unconjugated meningococcal polysaccharide vaccine in subjects 56–65 years of age (Stamboulian et al., 2010). Finally, a single dose of MenACWY-CRM was found to induce a robust immune response against all four meningococcal serogroups in a study of 180 Indian subjects, although only one third of the population was aged 19–75 years, and older individuals were not analyzed separately (Lalwani et al., 2015).

Regarding travellers, the ACIP recommends that travellers to African countries in the “meningitis belt” during the dry season (December–June) receive vaccination with a MenACWY vaccine before travel (Mbaeyi et al., 2020). Similarly, advisories recommending vaccination may be issued for travellers to other areas when outbreaks of meningococcal disease caused by vaccine-preventable serogroups are ongoing. The ACIP further recommends that international travellers receive a booster dose of MenACWY if the last dose was administered 3–5 or more years previously. Of note, meningococcal vaccination is required by the Kingdom of Saudi Arabia for all travellers to Mecca during the Hajj and Umrah pilgrimages (Mbaeyi et al., 2020).

### Typhoid Fever

Diseases with a fecal-oral route of transmission have a huge impact worldwide, and include typhoid, cholera and poliomyelitis (polio). Typhoid and paratyphoid fevers, together referred to as enteric fever, are caused by systemic infection with *Salmonella enterica* subspecies serovars Typhi and Paratyphi A, B, and C (Typhoid and Paratyphoid, 2019). It is estimated that more than 14 million cases of enteric fever occurred in 2017, with 76.3% of all cases caused by *Salmonella enterica* serotype Typhi. The global case fatality rate is estimated at 0.95%, corresponding to around 135,900 deaths from enteric fever annually, and is higher among older adults, and among those living in lower-income countries. The mortality rate is higher among patients aged over 50 years compared to those under 50 years of age (3.3 vs. 0.4%; *p* = 0.009), although these data date from epidemiological information collected from 1977 to 1979 regarding 901 cases of typhoid reported in the United States (Taylor et al., 1983). The majority of cases occur in South/South-East Asia, and sub-Saharan Africa (World Health Organization, 2018b). In travellers to destinations in South Asia, typhoid fever is the third most commonly contracted disease, after traveler’s diarrhea and influenza (Steffen et al., 2015a). In a study from the Swedish database on notifiable communicable diseases, including all cases recorded of typhoid and paratyphoid fevers from 1997 to 2003, Ekdahl et al. reported an overall risk of enteric fever after travel of 0.42 per 100,000 travellers (Ekdahl et al., 2005). The highest risk was observed in travellers returning from India and neighboring countries (41.7 per 100,000), the Middle East (5.91 per 100,000), and Central Africa (3.33 per 100,000), while the risk was comparatively lower in East Asia (0.24 per 100,000) (Ekdahl et al., 2005).

Currently, there are several typhoid vaccines available, including the unconjugated polysaccharide vaccine, containing purified Vi capsular polysaccharide from the Ty2 *S. Typhi* strain; and the Ty21a vaccine, which is an orally administered live attenuated Ty2 strain of *S. Typhi* modified by chemically-induced mutagenesis (World Health Organization, 2018b). Typhoid conjugate vaccines (TCV) have also been developed, consisting of a Vi polysaccharide antigen linked to various carrier proteins. Three such TCVs have been licensed to date, all in India, and others are in the pipeline (Syed et al., 2020). No TCV is licensed in Europe at present. Studies of typhoid vaccines to date have been performed mainly in countries with high endemicity, and not specifically among travellers, which may represent a drawback to estimating vaccine efficacy for travellers. Nevertheless, the WHO recommends vaccination for travellers from non-endemic to endemic areas, in addition to adherence to precautions on hygiene practices (World Health Organization, 2019b).

The conjugate vaccine is a single-dose, intramuscular injection, with an estimated efficacy of 87% when evaluated in a human challenge study in a population of 112 immunologically naive adult (age 18–60 years) volunteers (Jin et al., 2017). There are no head-to-head comparisons of TCV vs. the live attenuated Ty21a vaccine, and the potential for a herd effect with TCV has never been investigated (World Health Organization, 2018b). Evidence suggests that protection lasts up to 5 years after the first dose (Voysey and Pollard, 2018), but data are lacking regarding the need for booster doses.

The polysaccharide vaccine is administered in a single dose, either intra-muscularly or subcutaneously, and is recommended for adults. Immunity begins to wane after 2 years in vaccinated subjects, and hypo-responsiveness to revaccination has been reported in some studies (Keitel et al., 1994; Overbosch et al., 2005; Zhou et al., 2007; Roggelin et al., 2015), but existing data are not sufficiently robust to suggest a risk of hypo-responsiveness (World Health Organization, 2018b). The increased risk of infection with typhoid fever observed beyond 2 years after vaccination underpins the recommendation to re-vaccinate every 3 years in order to maintain protection.

The live attenuated Ty21a is given on a 3 dose schedule, by the oral route, ingested on days 1, 3 and 5. It stimulates serum and mucosal antibodies, eliciting long-lived cell-mediated immune responses (World Health Organization, 2018b). There is also evidence to indicate that it induces herd protection (Levine et al., 1989), which is useful from the public health perspective. Protective efficacy of up to 67% was reported at 3 years after vaccination, and 62% after 7 years (World Health Organization, 2018b). Revaccination is recommended every 3 years forViPS, and every 3–7 years for Ty21a (World Health Organization, 2019b). The need for revaccination with TCV is currently unclear (World Health Organization, 2019b). The Ty21a vaccine is well tolerated and associated with low rates of adverse events. However, the live attenuated vaccine should not be given in immunocompromised patients, including those taking immunomodulators, calcineurin inhibitors, cytotoxic agents, antimetabolites, and high-dose steroids (Centers for Disease Contr, 2020b).

In view of the persisting high burden of typhoid fever and the emergence of antimicrobial resistant strains of *S. Typhi*, the WHO recommends programmatic use of typhoid vaccines for the control of typhoid fever from a public health perspective, and recommends the TCV as a 0.5 ml single dose in adults up to 45 years in typhoid endemic regions. Concerning travellers from non-endemic to endemic areas, vaccination with any of the available licensed vaccines is recommended, in conjunction with usual water and sanitation hygiene precautions. Certain special populations who may be at increased risk of acquiring or transmitting *S. Typhi* infection should also be targeted for vaccination, such as professional food handlers working in endemic areas, or healthcare and laboratory workers.

Certain precautions should also be noted regarding typhoid vaccination. Firstly, there are currently no data on the interchangeability or sequential use of the different available vaccines. Second, typhoid vaccines are contraindicated for individuals with known hypersensitivity to any component of the vaccine. Third, the polysaccharide vaccine can be co-administered with other vaccines routinely recommended for international travellers, such as YF and hepatitis A vaccines (World Health Organization, 2018b). Certain antimalarials particularly mefloquine, exhibit activity against Ty21a, and may suppress the Ty21a antibody response, and should therefore not be given from 3 days before until 3 days after giving the Ty21a vaccine (World Health Organization, 2018b). Antimicrobial agents may also be active against the typhoid strain in the vaccine, and therefore, the typhoid vaccine should be delayed by at least 72 h after antimicrobial administration. Fourth, *Salmonella paratyphi A* and *B* do not have the Vi antigen, and thus, the polysaccharide vaccine is not effective against these forms of infection (Guzman et al., 2006; Fangtham and Wilde, 2008). Finally, data are sparse regarding seroconversion rates in older individuals after vaccination.

### Cholera

Cholera is an acute diarrheal disease in humans caused by the Gram-negative bacillus *Vibrio cholerae*. Cholera can be contracted *via* the fecal-oral route, or from the environment, and is characterized by the abrupt onset of acute watery diarrhea and vomiting. Prompt treatment with rehydration solution and antibiotics is highly effective in treating cholera, but if left untreated, severe cases can rapidly result in death (Ganesan et al., 2020), with death rates up to 70% reported (Ramamurthy et al., 2019), although the majority of infected individuals will only suffer mild diarrhea, or may even be asymptomatic (Jilg, 2020). The estimated incidence is between 1.3 and 4.0 million cases per year, with up to 143,000 deaths (Ganesan et al., 2020). Modern sanitation has led to the virtual eradication of cholera in industrialized countries, with disease now mainly occurring in low-resource countries with poor infrastructure, especially in terms of sanitation and access to clean drinking water (Rabaan, 2019). A global strategy to control cholera was launched by the WHO in 2017. The mainstay of prevention of both endemic cholera and outbreaks is the provision of safe drinking water and adequate sanitation, while cholera vaccination can be implemented as a complementary measure. Published data about the risk of cholera in travellers is scarce, although a recent systematic review of 91 reports of cholera or diarrhoea among travellers from 1990 to 2018 found only 156 cases of cholera imported as a consequence of travel, and cholera vaccination information was not provided in most of the reports (Connor et al., 2019).

There are currently two types of oral cholera vaccine available internationally for global use, namely WC-rBS, killed whole cell monovalent (O1 subtype) vaccines with a recombinant B subunit of cholera toxin, and second, WC, killed modified whole cell bivalent (O1 and O139) vaccines without the B subunit (World Health Organization, 2018c). In addition, a single-dose oral live attenuated vaccine is approved in the United States only for use among travellers to areas with active cholera transmission, namely CVD 103-HgR (Wong et al., 2017). It was shown to have vaccine efficacy of 90.3% at 10 days, and 79.5% at 3 months in a human cholera challenge model among 197 healthy adult volunteers aged 18–45 years old (Chen et al., 2016). In a summary of opinion dated January 30, 2020, the Committee for Medicinal Products for Human Use of the European Medicines Agency (EMA) recommended marketing authorization for this new cholera vaccine (European Medicines Agency, 2020). Limitations of this vaccine at present include the fact that its effectiveness has not been established in persons living in cholera-affected areas, nor in those who have pre-existing immunity due to previous exposure to *V. cholerae* or receipt of a cholera vaccine. Similarly, CVD 103-HgR has not been shown to protect against disease caused by *V. cholerae* serogroup O139 or other non-O1 serogroups. Reported side effects include tiredness, headache, abdominal pain, nausea/vomiting and loss of appetite (Chen et al., 2016). Finally, it should be remembered that cholera vaccination offers incomplete protection, and must always be considered as a complementary measure on top of standard water, sanitation and hygiene measures.

A common consideration for the use of cholera vaccine that is particularly relevant to older travellers is to prevent traveler’s diarrhea. Indeed, traveler’s diarrhea, caused by enterotoxigenic *Escherichia coli* (ETEC) frequently occurs in individuals traveling from industrialized countries to less industrialized zones. The whole-cell *V. cholerae* O1 in combination with a recombinant B-subunit of cholera toxin (WC/rBS) vaccine (Dukoral®) has been shown to provide cross-protection against traveler’s diarrhea caused by *E. Coli* (Leung et al., 2006; Jelinek and Kollaritsch, 2008; López-Gigosos et al., 2013). A Cochrane systematic review concluded that there was insufficient evidence to support the use of cholera vaccine to prevent traveler’s diarrhea (Ahmed et al., 2013), and another recent review concluded that no vaccine offers satisfactory protection (Steffen et al., 2015b). The recent guidelines for the prevention and treatment of traveler’s diarrhea from the International Society of Travel Medicine (Riddle et al., 2017) make no mention of vaccination for the prevention of traveler’s diarrhea, and stipulate that prophylaxis should only be considered in high risk groups, such as those with underlying health conditions, which may often be the case of older persons. Indeed, diarrheal diseases in older persons are often more serious, and patients with cardiovascular or kidney disease may tolerate dehydration poorly (Jilg, 2020). These considerations should be weighed on a case-by-case basis when considering the need for cholera vaccination in older individuals traveling to areas where cholera is endemic.

### Poliomyelitis

Poliomyelitis (polio) is a highly infectious viral disease that mainly affects children under 5 years of age. It invades the nervous system and leads to irreversible paralysis in 0.5% of cases. Polio has been successfully controlled in recent decades, and almost eradicated worldwide, with a decrease of over 99% in wild polio virus cases since 1988. However, wild polio viruses are still endemic in some areas, such as Afghanistan, Nigeria and Pakistan, and an outbreak recently occurred in Papua New Guinea (Hall et al., 2019). Until polio is completely eradicated, all countries remain at risk of imported wild type polio virus cases, and there could be as many as 200,000 new cases every year within 10 years all over the world. This led the WHO to propose the “Polio Endgame Strategy” (World Health Organization, 2019c), a comprehensive approach to working through the final challenges toward complete eradication, and laying the groundwork for a sustainable, polio-free future.

There are two facets to polio eradication, namely the wild polio virus and circulating vaccine-derived poliovirus. Vaccination is key to achieving eradication of polio, since the disease cannot be treated, but only prevented. The WHO goal is therefore to reach every last child with polio vaccination. Oral polio vaccines are the predominant vaccine used in the fight against polio, and contain one, two, or three serotypes of attenuated vaccine. They are inexpensive, safe, highly effective, and orally administered, meaning that no healthcare professionals or sterile equipment is required. Given that wild poliovirus type 2 was officially declared eradicated in 2015, wild type 2 has now been removed from the oral polio vaccine, since it was also responsible for nearly 90% of all cases of circulating vaccine-derived polio. In 2016, all countries switched to the bivalent oral polio vaccine, which contains only type 1 and type 3 components, thus greatly reducing the risk of vaccine-derived cases. The inactivated polio vaccine contains inactivated strains of all three serotypes, and is administered by intramuscular or intradermal injection. It must be administered by a trained healthcare professional. It elicits excellent protective immunity, and is up to five times more expensive than the oral vaccine. Insecure and inaccessible areas represent a major challenge to reaching populations for vaccination while at the same time maintaining front-line worker safety (World Health Organization, 2019c). For the long-term, future use of inactivated polio vaccine after wild-type polio transmission has been stopped will maintain population immunity levels (Bandyopadhyay et al., 2015).

Regarding travellers in particular, the WHO recommends that before traveling abroad, persons residing in countries with active transmission of a wild or vaccine-derived poliovirus should have completed a full course of polio vaccination in compliance with the national schedule, and received one dose of inactivated or bivalent oral polio vaccine within 4 weeks–12 months of travel (World Health Organization, 2017b). Certified polio-free countries may require incoming travellers from polio-infected areas to be vaccinated against polio to obtain entry. Travellers from polio-free to polio-infected areas should be vaccinated in compliance with their national schedule (World Health Organization, 2017b). The latest meeting of the Emergency Committee under the International Health Regulations (2005) (IHR) on the international spread of poliovirus underlined the ongoing frequency of WPV1 international spread and the increased vulnerability in countries where routine immunization and polio prevention activities have been adversely affected by the COVID-19 pandemic (World Health Organization, 2021). The committee issued temporary recommendations for polio-infected states with risk of international spread to require polio vaccination in residents and long-term (>4 weeks) visitors prior to undertaking international travel out of the country (World Health Organization, 2021). Most older individuals living in developed countries will have received polio vaccination during childhood, but one lifetime booster may be administered to previously-vaccinated adults who are at risk of increased exposure to polio virus, e.g., during travel [Prevention (2009). Update, 2009]. Adults who were partially vaccinated may receive the remaining doses of the schedule, regardless of the time interval since the initial doses were given [Prevention (2009). Update, 2009].

### Rabies

Rabies is a viral disease that causes fatal encephalitis. It is transmitted to humans *via* the saliva of an infected animal, and 99% of human cases are caused by bites from domestic dogs. Rabies causes an estimated 59,000 deaths per year worldwide (Hampson et al., 2015), and is almost invariably fatal once symptoms appear (Jackson, 2016). Rabies predominantly affects poor rural communities in countries where the disease is endemic (mainly Asia and Africa), and 40% of victims are children aged under 15 years of age (World Health Organization, 2018d). A prospective study of patients presenting to the rabies treatment center in Marseille over a 14-year period (1994–2007) reported a total of 424 injured travellers attending the center, with an increase in the number of patients over time (Gautret et al., 2010). The majority of cases were after travel to from North Africa (41.5%), where bites or scratches from cats and dogs accounted for the highest risk, and Asia (22.2%), where encounters with non-human primates carried the highest risk (Gautret et al., 2010).

With its extremely high case fatality rate, and given that rabies is 100% vaccine-preventable, containing and eradicating human dog-mediated rabies has become a major public health goal. In June 2018, the WHO in collaboration with the Global Alliance for Rabies Control launched the “Zero by 30: Global Strategic Plan” to end human deaths from dog-mediated rabies by the year 2030, an objective that is considered to be feasible with currently available technology and knowledge. To this end, awareness of the disease, how to prevent it, and how to act in case of a bite from a rabid animal, are all key points in achieving a world free of dog-mediated rabies.

The estimated incidence in travellers of a bite requiring post-exposure rabies prophylaxis (PEP) is 0.4 per 1,000 travellers per month (Gautret and Parola, 2012). However, even if the risk of rabies is low, the risk of an animal bite is high. Therefore, prevention is of paramount importance, through simple measures such as avoiding contact with street dogs, monkeys and other mammals. In case of a dog-bite, the wound should be washed immediately with soap and water, or flushed abundantly with water if no soap is available, and medical attention should be sought immediately in the nearest medical centre. This can be problematic for travellers in rural areas where medical help may not be easily available. For this reason, it is advisable to have travel insurance to ensure prompt assistance, especially for unvaccinated persons traveling to areas where rabies is endemic.

The severity of the bite from a suspected rabid animal determines the risk of rabies exposure, and thus, the type of PEP that needs to be implemented. The different categories of exposure are outlined in Table 3.

**TABLE 3:** Categories of exposure to rabies virus, according to World Health Organization (2018d).

Category	Description	Exposure level
Category I	Touching or feeding animals, animal licks on intact skin	None
Category II	Nibbling of uncovered skin, minor scratches or abrasions without bleeding	Exposure
Category III	Single or multiple transdermal bites or scratches, contamination of mucous membrane or broken skin with saliva from animal licks, exposures due to direct contact with bats	Severe exposure

Effective PEP should be provided after rabies exposure, consisting in thorough washing and flushing of the wound, prompt administration of rabies vaccine (if no pre-exposure prophylaxis was possible), and, in case of category III exposure, infiltration of human rabies immunoglobulin (HRIG) in and around the wound (Jackson, 2016). Obtaining HRIG may be difficult in many geographical areas, as the product is not readily available everywhere, is expensive, and needs to be administered rapidly (ideally within the first 2 days after the bite) for maximum efficacy. Recent studies indicate that only 5–20% of travellers who sustained category III exposure and who had an indication for HRIG actually received it in the country of exposure (Gautret et al., 2018). Similarly, in a study of 75 cases of rabies exposure treated with HRIG, Soentjens et al. observed a substantial time delay between exposure and administration of HRIG, in particular when the injury occurred abroad (Soentjens et al., 2019a). Therefore, promoting pre-exposure prophylaxis is a key preventive strategy for avoiding the risk of rabies, especially during travel to endemic areas. Rabies pre-exposure prophylaxis (PrEP) greatly simplifies PEP, by obviating the need for HRIG, and by reducing the number of PEP vaccine doses required (Soentjens et al., 2019b).

In April 2018, the WHO published new guidelines for rabies pre- and post-exposure prophylaxis. The new WHO recommendations for rabies vaccination are summarized in Table 4. Regarding PrEP, the WHO now recommends a 2-visit regimen, with the additional possibility of a single-visit shot for last-minute travellers who are leaving at very short notice. This new recommendation aims to increase cost-effectiveness, and to spare both doses and time, while still ensuring safety and clinical effectiveness (World Health Organization, 2018d).

**TABLE 4:** WHO recommendations for pre- and post-exposure rabies prophylaxis.

Pre-exposure prophylaxis			
-2-sites ID on days 0 and 7 OR -1-site IM on days 0 and 7			
**Pre-exposure prophylaxis under specific circumstances** ^a^			
-2-sites ID on day 0 OR 1-site IM on day 0			
**Post-exposure prophylaxis**			
	**Category I exposure**	**Category II exposure**	**Category III exposure**
Immunologically naïve, all ages	No PEP required	Immediate vaccination:	Immediate vaccination:
		-2-sites ID on days 0, 3 and 7	-2-sites ID on days 0, 3 and 7
		OR	OR
		-1-site IM on days 0, 3, 7 and between day 14–28	-1-site IM on days 0, 3, 7 and between day 14–28
		OR	OR
		-2-sites IM on days 0 and 1-site IM on days 7 and 21	-2-sites IM on days 0 and 1-site IM on days 7 and 21
		RIG is not indicated	RIG administration is recommended
Previously immunized, all ages	No PEP required	Immediate vaccination^b^:	Immediate vaccination^b^:
		-1-site ID on days 0 and 3	-1-site ID on days 0 and 3
		OR	OR
		-4-sites ID on day 0	-4-sites ID on day 0
		OR	OR
		-1-site IM on days 0 and 3	-1-site IM on days 0 and 3
		RIG is not indicated	RIG is not indicated

ID, intradermal injection; IM, intramuscular injection; RIG, rabies immunoglobulin.

a When there are time constraints or a single visit to the clinic is the only option, the shortened PrEP course can be given, but the patient should plan to receive a second vaccination as soon as possible to complete PrEP. In case of rabies exposure before the second vaccination, the patient is recommended to receive a full course of PEP, with RIG in cases of severe (Category III) exposure.

b Immediate vaccination is not recommended if complete PEP already received within <3 months previously.

Note: Immediate washing of the exposure surface/wound is recommended for all age groups, categories of exposure and types of prophylaxis.

As regards senior travellers in particular, there is a gap in the evidence concerning this population. In one retrospective study of vaccination data from 2,112 Belgian military personnel who received intradermal rabies PrEP with a three-shot regimen during the period 2014–2017, only one failed to achieve seroconversion to a level of rabies virus neutralizing antibodies (RVNA) >0.5 IU/ml, the threshold that is internationally recognized as safe and capable of being boosted with PEP vaccination (Van Nieuwenhove et al., 2019). However, most of the patients included in the study were younger than 60 years of age, and the oldest participant was 62. Data specifically in elderly patients are lacking. A recent systematic review and dose-response meta-analysis reported that older participants required a longer time to achieve maximum geometric mean titre (GMT) levels (42 days in older participants with a median age of 62 years, vs. 30 days in the group with a median age of 28 years) (Xu et al., 2021). In addition, the maximum level achieved by older participants was lower. In this study, no dataset was identified that contained information on GMT levels after PrEP in adults over 50 years of age (Xu et al., 2021). In another study assessing rabies-specific neutralizing antibody responses in a cohort of rabies vaccine recipients (including 97 subjects aged over 50 years) over a period of 20 years, Mansfield et al. reported that following a primary vaccination course, subject age did not appear to influence antibody response, although subjects in the older age group (>50 years) showed a significant decline in antibody titers over time (Mansfield et al., 2016). Finally, in a study of 835 travellers (37.1% aged >50 years) who received intra-dermal PrEP at a travel medicine clinic in South Australia from 2000 to 2016, Furuya-Kanamori et al. investigated immunogenicity post-primary course and post-booster (Furuya-Kanamori et al., 2020). They reported that among the 145 subjects who were aged 60 years or over, 90.5% (95% CI 83.9–94.5%) and 86.7% (95% CI 57.4–96.9) were seropositive after a primary course consisting of 3 doses of 0.1 ml (days 0, 7 and 21–28) or 2 doses of 0.1 ml (days 0 and 7) respectively. Furthermore, the respective rates of seropositive individuals in the group aged 60 years and over were 96.7% (95% CI 91.5–98.8) and 100% after a booster dose, even when the booster dose was given 3 years or more after the initial course, including in older individuals (Furuya-Kanamori et al., 2020).

In this context, the recommendation for rabies prophylaxis in senior travellers is to apply the same principles and procedures as for younger travellers. As stated in the 2018 WHO guidelines, pre- and post-exposure schedules are recommended for all ages. When considering PrEP, the duration of the stay in endemic areas, the remoteness of the destination, the type of activity planned (outdoor activities, caves etc.) the likelihood of prompt medical assistance (potentially including HRIG) being available, are all factors that should be taken into account.

### Japanese Encephalitis

Japanese Encephalitis (JE) is an infectious disease of the brain caused by the Japanese encephalitis virus (JEV), an arbovirus spread to humans by *Culex* mosquitoes. It is the most common viral encephalitis in Asia, occurring more frequently in children under the age of 15, because naïve, and in the elderly, as a possible consequence of diminished protection. However, in travellers arriving from non-endemic countries, it can obviously occur at any age since there is no previous immunity (Yun and Lee, 2014). The risk of JE for visitors to endemic areas is clearly dependent on the travel itinerary and exact destination and activities, and has historically been reported to be low, at less than 1 case per million travellers (Hills et al., 2010). However, more recent analyses have advanced the lower figure of 1/400,000 per visit (Lindquist, 2018). Moreover, the consequences of climate change, the spread of the disease into new areas and to both urban and peri-urban settings have made JE an unpredictable emerging disease (Lindquist, 2018).

There is no specific treatment for JE and therefore, and with roughly 30% of fatal cases and 30–50% of survivors with neurologic sequelae (Hills et al., 2019), besides protection from mosquito bites, vaccination remains the mainstay of prevention. There are different vaccines available worldwide, with varying distribution: the most common are an inactivated Vero cell vaccine (licensed in the United States, Europe, and several Asian countries), a live attenuated vaccine (licensed in all Asian endemic countries) and a chimeric live attenuated vaccine (licensed in Australia and a few other endemic countries). Seroconversion rates in older adults are about 65% with the inactivated vaccine available in Europe, whereas data from Australia show that the chimeric vaccine can be immunogenic in >99% of vaccinated individuals older than 60 years of age (Australian Government Dep, 2010). A single-centre, open label, phase IV trial comparing immune response to primary vaccination with the inactivated, adjuvanted JE vaccine reported that vaccine-specific antibody titres were significantly lower in older participants (mean 69, range 61–78 years, *n* = 30) and 47% of them were non- or low responders after the two doses of the vaccine neo-antigen (Wagner et al., 2018). Among 200 subjects aged 64–83 years, Cramer et al. observed seroconversion in 65% of subjects (95% CI 58.1–71.3%) at 70 days after vaccination with the Vero cell-amplified, purified, inactivated JE vaccine (Cramer et al., 2016). While all vaccines have a high profile of tolerability, live vaccines must be avoided in immune-compromised subjects, severe HIV patients as well as in pregnant and breastfeeding women.

### Tick-Borne Encephalitis

Tick-Borne Encephalitis (TBE) is another example of a viral infectious disease of the brain spread to humans through the bite of a vector, specifically hard ticks, most frequently of the genus *Ixodes*. Only very few cases of the disease are acquired through the consumption of unpasteurised dairy products. Affected areas spread from Central-Eastern Europe north and eastwards through Russia and Siberia up to the coastline of the Pacific Ocean. Publications of imported cases of TBE due to travel are scarce, and therefore, the travel-associated risk is difficult to identify, but based on estimates of exposed visitors, Steffen et al. roughly extrapolated the attack rate for an undefined period of time to be 0.5–1.3 per 100,000 (1 per 77,000–200,000) overall in Western/Central Europe endemic areas for the exposed at-risk population (Steffen, 2016). TBE is endemic in many countries of the European Union, including Germany, Austria, Sweden and Switzerland (European Centre for Disea, 2012), yet many travellers may not be aware that such destinations might require specific vaccination. Several European countries recommend TBE vaccination, including Austria, Finland, the Czech Republic, Latvia and Slovenia. Sweden also recommendations vaccination consisting of three doses during the first year (or four if aged 50 or over) followed by a first booster dose after three years, then every 5 years, regardless of age.

Although TBE infection tends to display seasonality, accordingly to the tick life cycle (spring to autumn), global climate change and the milder winters make occurrence of disease possible all year round. The incidence varies greatly between countries and between regions of the same country. Regarding TBE infection among travellers, both the number at risk and the number affected are far from being precise (Steffen, 2016), and therefore only a crude estimate can be presented (Chrdle et al., 2016). As in other similar viral infections, the case-fatality rate is between 0.5 and 10%, but the percentage of survivors suffering from chronic neurologic complications can be as high as 60% (Taba et al., 2017). The clinical course is more severe with increasing age, especially as a consequence of immune senescence (Kunze, 2016).

Similar to what has been described for JE, prevention of TBE is also based on the avoidance of tick bites and more importantly, on vaccination (Chrdle et al., 2016). Four different TBE vaccines are available (two European and two Russian); all are safe and the main contraindications are represented only by severe allergy to eggs or to any specific component. There is no upper age limit to receive the vaccine, especially in consideration of the worse clinical scenarios in older adults. Both European vaccines require three doses for the full primary schedule. Boosters are needed every 3–5 years depending on risk exposure and age (the older the vaccinee, the shorter the boosting interval). A shorter primary schedule is validated for both European vaccines (Haditsch and Kunze, 2013). In a systematic review evaluating the immunogenicity and safety of TBE vaccination, Rampa et al. reported that lower protective antibody titers were found, with a diminishing immune response in individuals aged 60 years and over, and in studies investigating vaccine failure, most (although not all) failures occurred in individuals aged 50 years or over (Rampa et al., 2020). In one of the studies included in this systematic review, Weinberger et al. investigated antibody titers and booster responses in 79 healthy Austrian volunteers receiving TBE vaccination and aged 50 years or over, in comparison to a control group aged <30 years (Weinberger et al., 2010). They reported that lower antibody concentrations and neutralizing titers were found starting from the age of 50 years, and that booster vaccination even 5–7 years after the previous vaccine administration was successful in inducing adequate protection, even in older individuals (Weinberger et al., 2010).

### Dengue

Dengue is the most widespread mosquito-borne disease after malaria, and the leading cause of fever in returning travellers, ahead of malaria (Schwartz et al., 2008). A Swedish study of residents with confirmed dengue after travel between 1995 and 2010 reported an increasing trend over time for most destinations, with the majority of dengue cases acquired in Thailand (492/925 travellers; 53%) (Rocklöv et al., 2014).

Dengue fever is caused by a flavivirus, named dengue virus, transmitted to humans by *Aedes* mosquitoes. This virus exists in 4 serotypes, each able to elicit a serotype-specific sustained immune response. According to the most accepted theory, antibodies arising from a first dengue infection act as an enhancer in case of a new infection with a different serotype, causing the novel virus to enter more rapidly into target cells and multiply faster. This in turn increases the risk of severe disease, especially the so-called dengue haemorrhagic fever (the theory of the “enhanced antibody response”) (Halstead and Wilder-Smith, 2019). The substantial climate changes of recent years, the wider distribution of *Aedes* mosquitoes, and the mounting evidence that *Aedes albopictus* (Asian tiger mosquito) is also becoming a better vector for this virus, all make dengue likely the most important arboviral infection on the international scene (Johansson et al., 2019). Although there have been few studies specifically in older people, available data suggest that the disease is of greater severity in older individuals, with worse outcomes compared to their younger counterparts (Lee et al., 2013; Rowe et al., 2014).

A call for a vaccine was launched several years ago, ending with the license of the first tetravalent dengue vaccine in 2015 (Villar et al., 2015). Shortly after its launch, evidence that the number of hospitalisations secondary to vaccination tended to increase in naïve children in the Philippines made the WHO withdraw the campaign of immunisation, and limit the use of this vaccine only to people with proven existing immunity to a previous dengue infection (Wilder-Smith, 2018). A new tetravalent dengue vaccine, presumably free from drawbacks that beset its predecessor, is about to be launched (Biswal et al., 2019), giving new hope for effective prevention of this scourge.

## Conclusion

In summary, the number of older people who have the financial resources and good health to travel is increasing dramatically, and age in itself is not a contra-indication to travel. However, age-related changes to the immune system and the higher prevalence of chronic disease and frailty create a specific risk profile for older travellers. All older individuals planning to travel should be advised during specific pre-travel consultations, or during routine visits to a healthcare professional, about travel-specific vaccines, and preventive behaviors in view of travel. The destination, duration and purpose of the trip, the activities planned, the type of accommodation and plans for other future travels are all important elements that will help orient advice and information prior to travel, while patient-specific characteristics, such as health status and current medications, must be noted to tailor specific recommendations. Senior travellers should consult a travel medicine specialist long in advance of a planned trip in order to allow time to obtain appropriate travel-specific vaccines. Physicians should allow ample time for consultations with elderly patients envisaging travel, as these patients may need more information, and their health status may require more thorough investigation.
